# Myrobalan Fruit Extracts Modulate Immunobiochemical Pathways In Vitro

**DOI:** 10.3390/antiox14030350

**Published:** 2025-03-17

**Authors:** Stefanie Hofer, Marcel Jenny, Angela Klein, Kathrin Becker, Lucia Parráková, Florian Überall, Markus Ganzera, Dietmar Fuchs, Hubert Hackl, Pablo Monfort-Lanzas, Johanna M. Gostner

**Affiliations:** 1 Institute of Medical Biochemistry, Medical University of Innsbruck, Biocenter, 6020 Innsbruck, Austria; 2 Institute of Pharmacy, Pharmacognosy, Center for Molecular Biosciences (CMBI), University of Innsbruck, 6020 Innsbruck, Austria; 3 Institute of Biological Chemistry, Medical University of Innsbruck, Biocenter, 6020 Innsbruck, Austria; 4 Institute of Bioinformatics, Medical University of Innsbruck, Biocenter, 6020 Innsbruck, Austria

**Keywords:** myrobalan fruits, antioxidants, inflammation, immunobiochemistry, cell models, gene expression analysis

## Abstract

Myrobalan fruits are important ingredients of traditional remedies, such as the Ayurvedic formulation Triphala or the Tibetan formulation Bras bu 3. Myrobalan-containing remedies are described to have positive effects on metabolism, the cardiovascular system, and the immune system. The chemical composition of botanical mixtures can be very complex, and it is often impossible to identify individual compounds as specific active ingredients, which suggests a multi-target mode of action. In this in vitro study, the effect of myrobalan extracts in human cell models was investigated to gain more information about the molecular mechanism of action and to find possible synergistic effects. Direct and indirect antioxidant effects were investigated, and the activation of immunobiochemical metabolic pathways involved in the cellular immune response was examined in cell lines treated with extracts of the fruits of *Phyllanthus emblica*, *Terminalia chebula* and *Terminalia bellirica*, as well as a combination of them. In particular, a synergistic effect on the activation of the endogenous antioxidant defence system was observed with the combined treatment of the three fruit extracts. An integrated transcriptome analysis of cells treated with a combination of fruit extracts confirmed an effect on immune pathways, oxidative stress, and detoxification processes. This study shows the modulation of various signalling pathways and cellular processes that may be part of the multi-target mechanism of individual and combined myrobalan fruit extracts. Although the results are limited to in vitro data, they contribute to a better understanding of how botanical mixtures work and provide hypotheses for further research.

## 1. Introduction

In recent times there has been an increasing interest to decipher the molecular mode of action of traditionally used botanical preparations. Due to the dysregulated redox homeostasis associated with several diseases, major attention has been paid to investigate the antioxidant capacities of phytochemicals. However, it has become apparent that in several cases, it is not a sole active principle component but the concerted action of a plethora of phytochemicals that are relevant for the mode of action [1]. Moreover, interference of these mixtures with cellular signalling events leads to changes that go far beyond monocompound-mediated effects [2,3]. With respect to the multitude of synergistic, antagonistic, and additive effects of the several thousands of compounds contained in a botanical remedy, we and others suggested earlier to investigate the mode of action of botanical multi-components in an unbiased manner by a combinatorial approach of transcriptional analysis and pathway/target-based strategies [3,4,5].

Myrobalan fruits, which are contained in remedies such as the traditional Tibetan remedy ‘Bras bu 3’ (BB3), known in Ayurveda as ‘Triphala’, are used to treat a variety of conditions, such as fevers, cough, stomatitis, digestive problems, poor food absorption, or high blood pressure. The formulation is composed of three myrobalan fruits, i.e., *Terminalia bellerica* (beleric myrobalan), *Terminalia chebula* (chebulic myrobalan), and *Phyllanthrus emblica* (ebolic myrobalan), also known as amla [6,7]. While in Ayurveda, the corresponding formula Triphala (= three-fruit formula) is used in a 1:1:1 ratio usually, in the system of Tibetan medicine, for the formula BB3, the ratio 1:2:1 is more frequent. However, for both traditional medical systems, different proportional recipes are also mentioned in the literature. Respective commercial products are available in the Western world as dietary supplements.

All three myrobalan fruits are very rich in tannins (up to 40%), such as chebulic acid, chebulinic acid, and gallic acid (*T. bellerica* and *T. chebula*), as well as emblicanin A and B and pedunculagin, which also explain the bitterness of *P. emblica* [8,9]. Flavonoids like kaempferol are constituents of this plant, too [10].

The reported antidiabetic, cardiovascular, and hepatoprotective activities of Triphala [7,8,10] can be attributed to the presence of these phenolic compounds [11]. Moreover, they exert pronounced antioxidant and anti-inflammatory effects by directly scavenging different types of radicals like, e.g., hydroxyl radicals, superoxide anion, and nitric oxide, and by reducing lipid peroxidation [11]. Their indirect antioxidant properties derive from stimulating the activity of antioxidative enzymes like superoxide dismutase, catalase, glutathione-S-transferase, and glutathione peroxidase [12].

Changes in oxidative stress levels are essential for the induction, modulation, and termination of inflammatory as well as regenerative processes [13,14]. Reactive oxygen species (ROS) are key players in the tight regulation of these fine-tuned pathways that are essential for an effective immune response. However, if control mechanisms are absent or an imbalance of the redox homeostasis remains over a longer period of time, pathological conditions may develop [15].

For example, nuclear factor kappa B (NF-κB) is induced strongly by oxidative stress and is tightly controlled by positive and negative modulatory mechanisms. The transcription factor triggers the expression of chemokines and cytokines and thereby makes an important contribution to the orchestration of several immunological pathways. In addition, NF-κB is also involved in stress response mechanisms, in the regulation of cell survival and apoptosis, as well as in the modulation of differentiation, although its effects are very cell-type dependent and context-specific [16]. Another transcription factor that is induced strongly in response to oxidative stress and inflammation is the nuclear factor–erythroid 2-related factor 2 (Nrf-2). It regulates the antioxidant response element (ARE)-mediated expression of antioxidant enzymes that are important for the detoxification, reduction, and elimination of oxidized molecules [17]. One of those detoxifying enzymes is the heme oxygenase-1 (HMOX1), which is thought to influence both innate and adaptive immune responses by resolving early events in inflammation and thus minimizing the resulting tissue damage. Heme oxygenase is responsible for the degradation of the iron-containing, pro-oxidant heme to carbon monoxide (CO), biliverdin, and free iron. Biliverdin and its derivative bilirubin are known to be potent endogenous antioxidants, and CO exerts anti-inflammatory effects. Iron, on the other hand, exhibits pro-oxidant effects, but it induces the expression of the iron-storing protein ferritin, which acts an antioxidant itself [18,19].

Two important immunobiochemical pathways that are activated in the course of cellular immune activation are tryptophan (Trp) breakdown via indoleamine 2,3-dioxygenase (IDO-1) and the formation of the oxidative stress marker neopterin via GTP-cyclohydrolase I (GTP-CH-I) [12,13]. The breakdown of the essential amino acid Trp is a protective mechanism against invading pathogens by reducing the amount of Trp available for the reproduction of the microorganism. Reduced Trp concentrations and the formation of bioactive catabolites were shown to decrease T-cell proliferation and differentiation [20,21,22]. Elevated Trp breakdown can be observed in a variety of chronic diseases like infectious, autoimmune, cardiovascular, neurodegenerative, and neuropsychiatric, as well as malignant diseases. The increased ratio of kynurenine (Kyn) to Trp (Kyn/Trp) in the blood of patients is a surrogate marker for increased IDO-1 activity [23]. In addition, increased levels of neopterin can often be measured in these patients, too, as both pathways are activated in parallel during cellular (Th1-type) immune activation, mainly by interferon gamma (IFN)-γ [24]. Being closely related to the symptomatology and disease state, the two parameters, Kyn/Trp and neopterin concentration, are frequently applied as biomarkers for disease monitoring and are interesting immunobiochemical targets when studying the effects of (phyto)chemicals, food additives, and botanical mixtures [25,26].

The aim of this in vitro study was to investigate the effects of the botanical remedy BB3 and its fruit extract components (*Phyllanthus emblica fructus* (PEF), *Terminalia chebula fructus sine semine* (TCF), and *Terminalia bellirica fructus* (TBF)) on the previously mentioned immunobiochemical and cytoprotective pathways in immune and liver cell lines and to perform an integrated transcriptome analysis to decipher additional cellular targets that help to explain the mode of action.

## 2. Materials and Methods

### 2.1. Preparation of Ethanol Extracts

Pure powder of PEF, TCF, TBF, and BB3 and a mixture of the 3 fruit powders (in a ratio of 1:2:1) were kindly provided by PADMA AG (Wetzikon, Switzerland). Quality control included the determination of the tannin content, expressed as pyrogallol, in the single plant powders (PEF = 11%, TCF = 23%, and TBF = 11%) and of the tannin content and the total polyphenol content in the mixture (15% and 18.2%, respectively). Ethanol-water extraction was applied to efficiently extract bioactive compounds of different polarities [27]. The dried plant powders (5 g) were extracted using 25 mL of 70% ethanol ((*v*/*v*), Sigma-Aldrich, Vienna, Austria) at room temperature. After 24 h, the solution was centrifuged at 4000× *g* for 30 min to remove any debris. The sterile filtered extracts of PEF, TCF, TBF, and BB3 were stored light protected at room temperature. The final concentration of the extract was determined in 1 mL aliquots in triplicate by weighing the remaining mass, after removal of the solvent by vacuum evaporation using a rotary evaporator at 40 °C. The final concentration of the extract used for analysis is indicated as mg dry weight (extract)/mL of solvent.

### 2.2. Cell Culture

The human monocytic leukaemia cell line THP-1 (Research Resource Identifier (https://rrid.site, accessed on 3 March 2025) RRID:CVCL_0006; DSMZ, Braunschweig, Germany) and THP1-Blue^TM^ cells (RRID:CVCL_X585; Invivogen, Toulouse, France) were cultured in Roswell Park Memorial Institute (RPMI) 1640 medium, which was supplemented with 10% (*v*/*v*) heat-inactivated foetal calf serum (FCS, Gibco—Thermo Fisher Scientific, Vienna, Austria) and 2 mM L-glutamine (Sigma-Aldrich, Vienna, Austria). The selection antibiotic Zeocin (Invitrogen, Vienna, Austria) was added to the THP1-Blue^TM^ medium at 200 µg/mL.

The human liver cancer-derived cell line HepG2 (RRID:CVCL_0027; DSMZ, Germany) was cultured in Dulbecco’s Modified Eagle’s Medium (DMEM, Gibco—Thermo Fisher Scientific, Vienna, Austria) supplemented with 10% (*v*/*v*) heat-inactivated FCS. The CellSensor^®^ ARE-bla HepG2 cell line (RRID:CVCL_KS12; Invitrogen—Thermo Fisher Scientific, Vienna, Austria) was cultured in DMEM containing 10% (*v*/*v*) dialyzed FCS (Invitrogen—Thermo Fisher Scientific, Vienna, Austria) and 0.1 mM each (1×) of non-essential amino acids (NEAA, Invitrogen—Thermo Fisher Scientific, Vienna, Austria). During a standard culture, 100 U/mL of penicillin, 100 µg/mL of streptomycin, and 5 µg/mL of blasticidin (all reagents from Invitrogen—Thermo Fisher Scientific, Vienna, Austria) were added to the CellSensor^®^ ARE-bla HepG2 medium.

All cells were maintained in an antibiotic-free medium during the experiments.

### 2.3. Oxygen Radical Antioxidant Capacity (ORAC) Assay

The peroxyl-radical scavenging capacities of extracts were determined with the ORAC assay [28], as previously reported [29]. In brief, the signal of the fluorescent probe fluorescein (AnaSpec, Fremont, CA, USA) decreases over time upon addition of the peroxyl-radical generator 2,2′-azobis (2-amidinopropane) dihydrochloride (AAPH, Wako Chemicals, Neuss, Germany). Antioxidants delay the oxidation of fluorescein. Trolox, a water-soluble analogue of vitamin E (Sigma-Aldrich, Vienna, Austria), was used as a reference compound. The final ORAC values were expressed as μmol Trolox equivalents per gram of dried powder (μmol TE/g).

### 2.4. Measurement of NF-κB Activity in THP-1-Blue^TM^ Cells

THP-1-Blue^TM^ cells are reporter cells derived from the myelomonocytic cell line THP-1. THP-1-Blue^TM^ cells contain an NF-κB/activator protein-1 (AP1)-inducible promoter to activate the expression of the reporter secreted embryonic alkaline phosphatase (SEAP). Upon stimulation of the Toll-like receptor (TLR) with lipopolysaccharide (LPS), NF-κB signalling is activated, leading to the expression of SEAP and its subsequent secretion into the medium.

THP-1-Blue^TM^ cells were treated with medium; vehicle (0.4% % (*v*/*v*) EtOH); or increasing concentrations of BB3 and its single components (TCF, TBF, and PEF). After 30 min of incubation, cells were either left untreated or stimulated with LPS (1 µg/mL) (Sigma-Aldrich, Vienna, Austria) and incubated for 6 or 24 h. Secretion of SEAP into the supernatant was quantified using the QUANTI-Blue assay kit (QUANTI-Blue^TM^, Toulouse, Invivogen, France). The signal was measured at 635 nm with a spectrophotometric plate reader (Bio-Tek Instruments, Bad Friedrichshall, Germany).

### 2.5. Measurements of Tryptophan (Trp), Kynurenine (Kyn), and Neopterin

Trp and the Kyn concentrations were measured in the supernatant of THP-1 cells 48 h after treatment with different concentrations of BB3 and its single components (5–50 µg/mL), or a vehicle control, with or without the addition of LPS (1 µg/mL). High-pressure liquid chromatography (HPLC) analysis was performed according to Widner et al., with minor modifications [30,31]. 3-Nitro-L-tyrosine (Sigma-Aldrich, Vienna, Austria) served as an internal standard. The Kyn/Trp, an estimate of IDO-1 activity, is shown in µmol Kyn per mmol Trp. Neopterin concentrations were determined with a commercially available enzyme-linked immunosorbent assay (ELISA) (Thermo-Scientific BRAHMS, Hennigsdorf, Germany), according to the manufacturers’ instructions.

### 2.6. Nrf-2/ARE Reporter Gene Assay

In order to evaluate the interference of BB3 and its components with the Nrf-2-mediated stress response, the CellSensor^®^ ARE-bla HepG2 cell line was used. In this cellular reporter system, the expression of the bacterial β-lactamase gene is controlled by a cis-acting ARE response element, which can be induced by the activation of the corresponding endogenous transcription factor Nrf-2. The expression of β-lactamase can be determined by enzyme-mediated cleavage of the fluorescence resonance transfer (FRET) substrate [32]. 

CellSensor^®^ ARE-bla HepG2 cells were seeded in a 96-well plate (7.5 × 10^4^ cells/90 µL/well) and incubated for 8 h. Cells were either left untreated or treated with sulforaphane (25 µM) (Sigma-Aldrich, Vienna, Austria) as a positive control or BB3 and its components (25–200 µg/mL). Eighteen hours after treatment, cells were loaded with the LiveBLAzer™-FRET B/G substrate (CCF4-AM; Invitrogen—Thermo Fisher Scientific, Vienna, Austria), according to the manufacturer’s protocol. Fluorescence intensity (excitation/emission at 414 nm/460 nm and at 414 nm/538 nm) was measured with a Fluoroskan Ascent FL plate reader (Thermo Labsystems, Philadelphia, PA, USA).

### 2.7. Measurement of Intracellular ROS 

Relative changes of intracellular ROS levels in HepG2 cells were monitored using the probe 2′,7′-dichlorofluorescin diacetate (DCFH-DA) as a substrate. In brief, upon uptake, DCFH-DA is deacetylated by intracellular esterases to non-fluorescent 2′,7′-dichlorofluorescin (DCFH), which cannot pass cell membranes. In the presence of ROS, DCFH is oxidized to highly fluorescent 2′-7′-dichlorofluorescein (DCF) [33,34]. 

HepG2 cells (6 × 10^4^ cells/100 µL medium/well) were seeded in 96-well plates and incubated for 24 h. Then, cells were washed twice with phosphate buffered saline (PBS), followed by a treatment with 25 μM of DCFH-DA for 1 h, as described previously [4]. Subsequently, quadruplicate wells were either left untreated or treated with a solvent (0.3% (*v*/*v*) EtOH) or increasing concentrations of BB3 and its components (12.5–200 µg/mL) for 1 h. Thereafter, the cells were treated with 600 μM of the peroxyl radical generator AAPH (Wako Chemicals, Neuss, Germany). The fluorescence of DCF (excitation/emission at 485 nm/535 nm) was determined after 45 min with a Fluoroskan Ascent FL plate reader (Thermo Labsystems, Philadelphia, PA, USA).

### 2.8. Cell Viability Assay

To measure the viability of cells after treatment with BB3 and its single components, the CellTiter-Blue™ (Promega, Walldorf, Germany) reagent was added to the culture medium after 24, 48, or 72 h (final concentration in the culture medium was 10% (*v*/*v*) CellTiter-Blue™). Cells were incubated at 37 °C for 4 h. The fluorescence at 544 nm/590 nm was determined using a Fluoroskan Ascent FL plate reader (Thermo Labsystems, Philadelphia, PA, USA).

### 2.9. Statistical Analysis

The data are expressed as means ± standard deviation (SD) or ±standard error of the mean (S.E.M.). A statistical analysis was performed using the IBM Statistical Package for the Social Sciences (SPSS) 18.0 statistical software for Mac OS (Chicago, IL, USA). Data were analysed using a 1-way analysis of variance (ANOVA). The homogeneity of variance was tested by use of the Levene test. The Bonferroni test for multiple comparisons was used for post hoc analysis of the data with homogenous variances, while Tamhane’s post hoc analysis was used for datasets with nonhomogeneous variances. *p*-values of less than 0.05 were considered to be statistically significant.

### 2.10. RNA Extraction

THP-1 cells were treated with LPS (1 µg/mL) plus BB3 extract (25 µg/mL and 50 µg/mL) or LPS plus a vehicle control for 24 h. Cells were harvested by centrifugation, and the total RNA was isolated using the RNeasy Mini Kit (Qiagen, Hilden, Germany), including a DNase digest step (Qiagen, Hilden, Germany). The RNA was quantified at 260 nm and the ratio of the absorbance at 260 and 280 nm was used to assess the purity. The quality of total RNA was controlled by gel electrophoresis.

### 2.11. Reverse Transcription Quantitative PCR (RT-qPCR)

RT-qPCR analysis was performed in a total volume of 10 μL, using 12 ng of cDNA, 3 mM of magnesium chloride, 400 nM of specific primers, and the SensiFAST SYBR Lo-ROX One-Step kit (Bioline Reagents, London, UK) on a Rotor-Gene-Q 5plex HRM (Qiagen, Hilden, Germany) under the following conditions: initial denaturation at 95 °C for 60 s; 40 cycles: 95 °C for 15 s and 60 °C for 30 s (fluorescence acquisition); melt curve analysis: ramp from 60 to 95 °C (rising by one degree each step); 25 °C for 60 s. The efficiency (E) for each primer was estimated with serial dilutions of cDNA, and the relative expression ratio (R) of a target gene was calculated using following formula: R = (E target ^∆Ct target (control-treatment)^)/(E ref ^∆Ct ref (control-treatment)^), according to Pfaffl et al. 2001 [35]. E target indicates the real-time PCR efficiency of the target gene transcript; E ref indicates the real-time PCR efficiency of the reference gene transcript. The threshold cycle (Ct) is defined as the point at which the fluorescence rises significantly above the background fluorescence. The REST software ©2009 (by Corbett Research Pty Ltd./Qiagen group and M.W. Pfaffl) was used to test for significance.

### 2.12. Transcriptomics

RNA from three biological replicates were pooled to obtain a “biological average” of stimulated gene expression changes, as such an approach was proven to be feasible in earlier studies [4]. A microarray experiment was performed to study gene expression in treated and control groups. Gene expression analysis was performed using Affymetrix Human Genome U133 Plus 2.0 Arrays (Affymetrix, Santa Clara, CA, USA). All data preprocessing was performed in R version 4.2.0 (R Development Core Team 2008). In order to calculate absolute expression values, the Robust Multichip Average (RMA) normalization from the ‘affy’ package of R version 1.74.0 was used for background correction, normalization, and summarization. Relative log2 ratios were calculated by subtracting the preprocessed absolute expression values of the control group from the treatment group. An absolute log2 ratio threshold higher than 0.6 (|fold change| > 1.5) was used. Microarray data were deposited at Gene Expression Omnibus (accession number: GSE273987).

### 2.13. Functional Analysis

Reactome pathway enrichment was analysed using Over-Representation Analysis (ORA) and confirmed with Gene Set Enrichment Analysis (GSEA) via the ReactomePA R package (v1.40.0) [36]. Pathways with 15–1200 genes and an adjusted *p*-value < 0.05 (Benjamini-Hochberg correction) were considered significant. Additionally, Reactome pathway representation was performed using ClueGO (v2.5.9), in Cytoscape (v3.10.1), to visualize the network of the most relevant pathways [37].

## 3. Results

### 3.1. Antioxidant Capacity

Parts of the biological activity of plant extracts are often attributed to the antioxidative capacity of the contained phytochemicals. Therefore, the aqueous ethanolic myrobalan fruit extracts and their mixture with BB3 were assessed in the Oxygen Radical Absorbance Capacity (ORAC) assay, an in chemico test system that measures the decrease in fluorescence caused by an antioxidant in the presence of an free radical initiator. In this cell-free test system, the ability of compounds and extracts to counteract peroxyl-radical formation by breaking radical chain reactions through hydrogen transfer can be determined.

**Figure 1:**
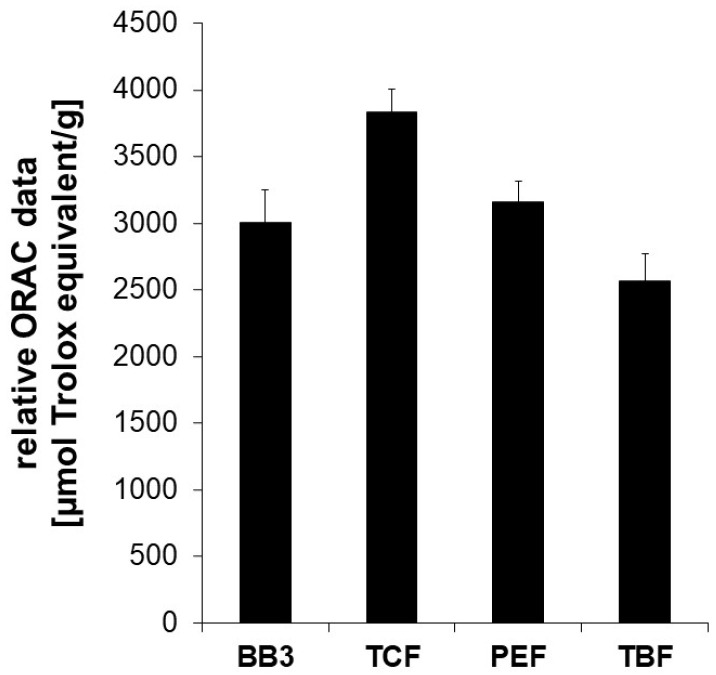
The oxygen radical absorbance capacity (ORAC) of myrobalan extracts and their mixture with BB3. The ORAC was determined in a cell-free system based on the inhibitory potential of the extracts on peroxyl-radical induced fluorescein decay. The water-soluble vitamin E Trolox was used as a reference. Relative ORAC values are expressed as µmol Trolox equivalents/g of dried extract. The shown results are the mean values ± S.E.M. of four independent experiments conducted in duplicates.

The extract of the BB3 mixture showed potent peroxyl-radical scavenging properties exhibiting an ORAC value of 3009 +/− 239 µmol TE/g. This means that 1 g of dried extract exerts the same oxygen radical absorbance capacity as 3009 +/− 239 µmol of Trolox. Among the single constituents of BB3, the dried extract of PEF showed the strongest oxygen radical absorbance capacity (3585 +/− 285 µmol TE/g), followed by TCF (3300 +/− 135 µmol TE/g) and TBF (2566 +/− 203 µmol TE/g) (Figure 1).

These results underline the antioxidant capacity of contained phytochemicals. However, as results from this and similar assay systems are frequently discussed regarding their relevance for living systems due to differences in pH, or unreflected bioavailability, further cell-based experiments were performed.

### 3.2. Effects of BB3 and Constituent Extracts on NF-κB Activity

**Figure 2:**
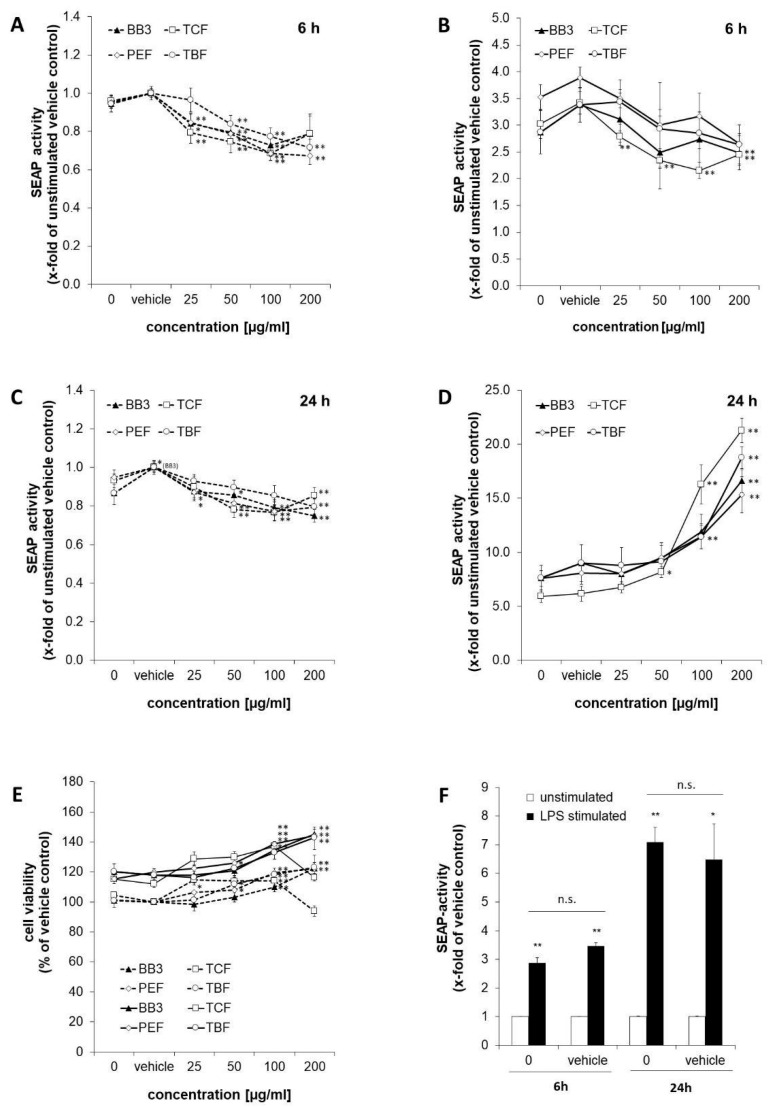
NF-κB activation in THP1-Blue^TM^ cells treated with Bras bu 3 (BB3) and its constituent myrobalan fruit extracts (TCF, PEF, and TBF). Cells were either left unstimulated (dashed lines) or stimulated with lipopolysaccharide (LPS) (continuous line). SEAP activity was measured to indicate NF-κB activation after 6 h (**A**,**B**) and after 24 h (**C**,**D**) of incubation. (**E**) Cell viability of THP1-Blue^TM^ cells 24 h after treatment with plant extracts. (**F**) Comparison of SEAP activity as a measure of (NF)-κB activation in cells treated with or without vehicle control (EtOH 0.35% (*v*/*v*)) after 6 or 24 h of LPS stimulation vs. no LPS treatment. Mean values ± S.E.M. of three independent experiments performed in triplicates are shown (* *p* < 0.05, ** *p* < 0.01, and n.s. = not significant).

SEAP levels in the supernatants of the reporter cell line THP1-Blue^TM^ were determined as a measure for NF-κB/AP-1 activity (Figure 2). Stimulation of the cells with LPS for 6 h augmented NF-κB activity by 2.9-fold compared to the untreated control cells (shown in Figure 2F). Similarly, in the vehicle control cells, stimulation with LPS led to an increase in SEAP activity of 3.5-fold. After 24 h, the effect of LPS was even stronger, and NF-κB/AP-1 activity was increased in the treated cells by 7.1- and 6.5-fold for the medium control and vehicle control cells, accordingly.

The effects of BB3 and its single components on NF-κB activity were studied in LPS-stimulated and unstimulated cells after 6 and 24 h. After 6 h, BB3 as well as its constituents led to a dose-dependent decrease in the unstimulated (Figure 2A) and even more in the LPS-stimulated cells (Figure 2B), respectively, with the most significant reduction being from 3.88-fold (stimulated vehicle) to 2.65-fold for 200 µg/mL of PEF extract in the stimulated cells. In the non-stimulated cells, the greatest inhibitory effect was also achieved by PEF in the highest concentration tested, and a reduction to 0.67-fold of the vehicle control was observed. The trend of a dose-dependent suppression of Nf-κB activation was present in all extracts with the 6 h treatment.

On the contrary, after 24 h, a significant and dose-dependent induction of NF-κB activity was observed for all of the plant extracts in the cells that had been stimulated with LPS (Figure 2D). For example, for the highest treatment concentrations of 200 µg/mL, the activity was induced up to 16.6-fold for BB3, up to 15.3-fold for PEF, up to 21.3-fold for TCF, and up to 18.8-fold for TBF, while the respective LPS-plus-vehicle treatments resulted in an activation in the range of 5.9- to 7.6-fold compared to the unstimulated vehicle control. In the unstimulated cells, however, NF-κB activity was slightly but significantly decreased by all of the different extract treatments (Figure 2C). Treatment with BB3 resulted in the strongest effect, with SEAP levels of 0.75-fold compared to the vehicle control.

Cell viability was assessed in the LPS-stimulated and unstimulated cells after 24 h of treatment by determining the metabolic activity with the probe resazurin (Figure 2E). Viability of the LPS-stimulated cells approximately increased by 1.2-fold compared to the non-stimulated cells. Treatment with plant extracts increased the viability under both LPS and non-LPS conditions, mostly in a dose-dependent manner and to a maximum of approximately 1.2-fold of the respective vehicle control. Only the treatment with 200 µg/mL of TCF led to a decrease in viability.

To summarize, the results show largely dose-dependent immunomodulating properties of all myrobalan extracts in terms of effects on NF-κB activity; however, the direction of perturbation was time-dependent, and effects were stronger in the LPS-stimulated compared to the unstimulated cells. The activities of the different extracts were similar, and no synergistic effect on NF-κB activity was observed in the BB3 mixture.

### 3.3. Effects of BB3 and Constituent Extracts on Tryptophan and Neopterin Metabolism

LPS stimulation leads to a parallel induction of Trp breakdown to Kyn and neopterin’s formation of THP-1 [38] and of NF-κB activation in THP1-Blue^TM^ cells [39] and previously has been used to investigate immunomodulatory effects of different compounds and nanoparticles.

**Figure 3:**
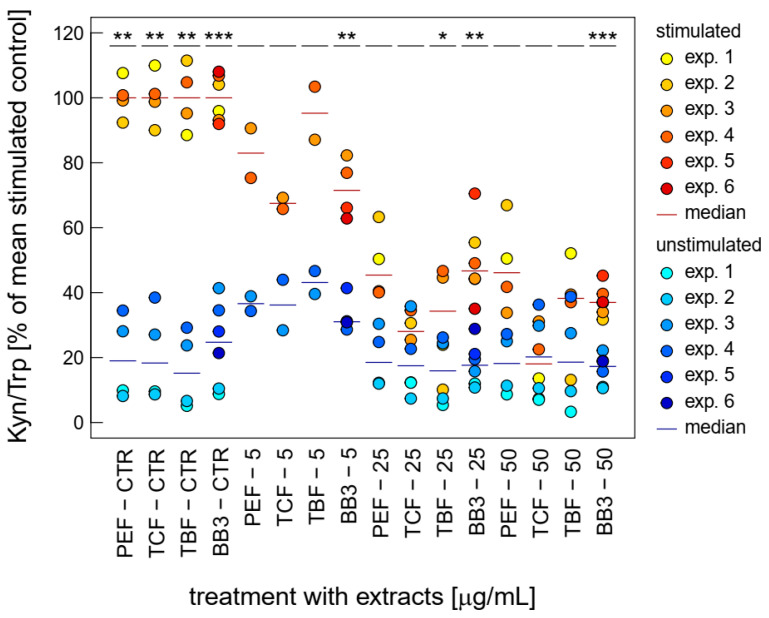
Effects of BB3 and its components in THP-1 cells after 48 h of treatment on the kynurenine to tryptophan ratio (Kyn/Trp), expressed as % of LPS-stimulated (1 µg/mL) controls. Extracts were tested in up to 6 experiments in stimulated (yellow to red) and unstimulated cells (blue). (* *p* < 0.05, ** *p* < 0.01, and *** *p* < 0.001).

Both LPS-stimulated Trp breakdown and neopterin formation is strongly inhibited by BB3 and the components of the natural remedy (Figure 3). The two closely related pathways seem to be major targets of the botanical mixture. Strong effects were already seen at relatively low concentrations (5 to 50 µg/mL) compared to the other bioactivities investigated in this study.

The single plant extracts and the BB3 mixture reduced the Kyn/Trp ratio, which serves as a measure for IDO-1 activity, significantly, in a dose-dependent manner in the LPS-stimulated THP-1 cells, almost back to base level, with maximum mean reductions of 67.4% (PEF), 87.2% (TCF), 76.1% (TBF), and 71.6% (BB3) in the highest tested concentration of 50 µg/mL (Figure 3). In the unstimulated cells, however, no significant changes could be observed with these treatment concentrations.

**Figure 4:**
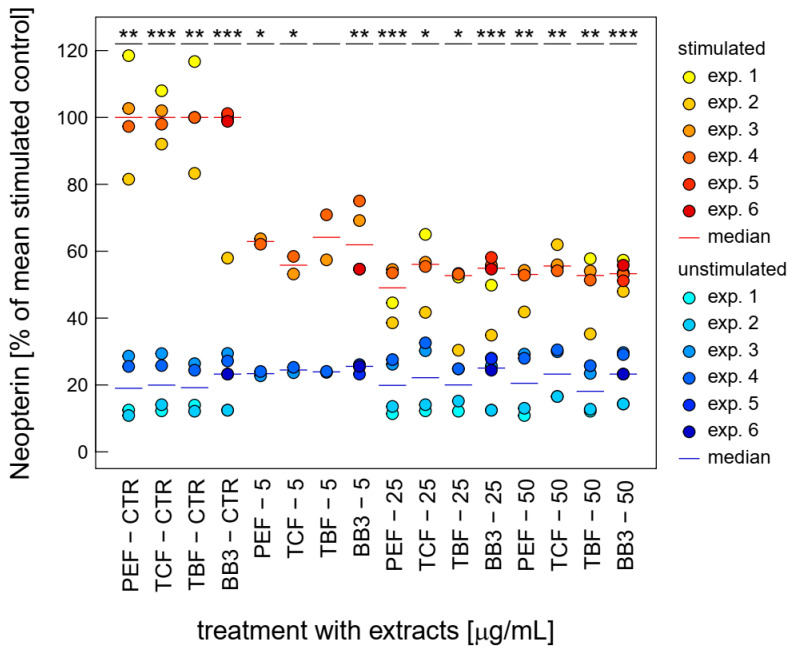
Effects of BB3 and its components in varying concentrations in THP-1 cells after 48 h of treatment on neopterin formation, expressed as % of LPS-stimulated (1 µg/mL) controls. Extracts were tested in up to 6 experiments in stimulated (yellow to red) and unstimulated cells (blue). (* *p* < 0.05, ** *p* < 0.01, and *** *p* < 0.001).

Similarly, neopterin formation was strongly inhibited upon treatment with BB3 and its single components at relatively low concentrations already. Over the whole tested concentration range, BB3 and the single myrobalan fruit extracts exhibited significant inhibitory effects on neopterin formation in the stimulated cells (as can be seen in Figure 4). The strongest reductions were observed at 25 µg/mL with a decrease of 84.7% (PEF), at 25 µg/mL with a decrease of 52.3% (TCF), at 25 µg/mL with a decrease of 78.6% (TBF), and at 25 µg/mL with a reduction of 45.4% (BB3).

### 3.4. Viability of HepG2 Cells

**Figure 5:**
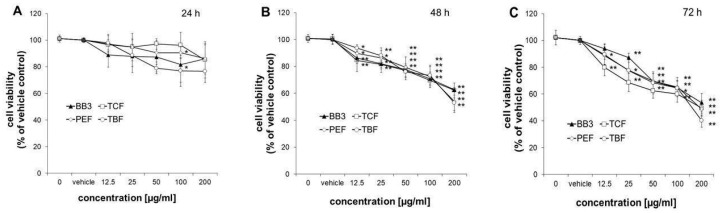
Effects of BB3 and the fruit extracts on cell viability. HepG2 cells were treated with vehicle (EtOH) or BB3 or myrobalan extracts (12.5–200 μg/mL) for 24 (**A**), 48 (**B**), and 72 h (**C**). Mean values ± S.E.M. of four independent experiments run in duplicates (* *p* < 0.05 and ** *p* < 0.005; compared to untreated cells) are shown.

The viability of HepG2 cells was measured after treatment with increasing doses of BB3, TCF, PEF, and TBF (12.5–200 µg/mL), extracted with 70% ethanol or the vehicle control (0.4% EtOH) for 24, 48, and 72 h. Treatment of cells for 24 h did not significantly affect their viability, with the exception of PEF and TBF at 100 µg/mL, which slightly decreased the number of viable cells to 90.6 ± 4.4% and 77.0 ± 11.7%, respectively (Figure 5A). After treatment of cells for 48 or 72 h, all extracts reduced the growth of cells already at doses of 12.5 µg/mL to 79.8–94.0% compared to the solvent control (Figure 5B,C). At the highest concentration tested (200 µg/mL), all extracts suppressed the growth of HepG2 cells in a similar manner to 52.9 ± 5.7–62.7 ± 1.8% after 48 h and to 40.1 ± 5.0–53.5 ± 6.9% after 72 h of treatment.

### 3.5. Cellular Antioxidant Activity in HepG2 Cells

**Figure 6:**
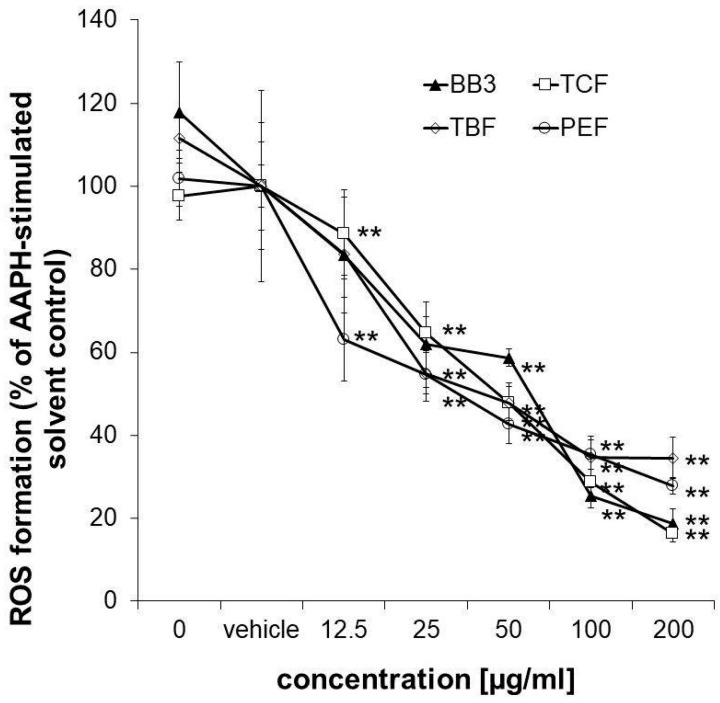
Measurement of intracellular ROS. Inhibition of peroxyl-radical (AAPH)-induced formation of ROS in HepG2 cells pretreated with increasing concentrations of BB3 or the myrobalan extracts (12.5–200 μg/mL). The mean percentages of DCF fluorescence, as a measure of ROS formation, are shown in relation to the AAPH-treated EtOH vehicle control (set to 100%). Mean values ± S.E.M. of three independent experiments run in quadruplicates (** *p* < 0.01; compared to AAPH-treated cells) are shown.

To confirm the antioxidant properties of the myrobalan fruit extracts, an antioxidant activity assay was performed in HepG2 liver cells. The applied assay protocol also takes the cellular bioavailability of the samples into account. All of the extracts but also the multi-component natural remedy showed strong and dose-dependent antioxidative properties in the tested concentration range (12.5–200 µg/mL), reaching a minimum of 27.88% (PEF), 16.23% (TCF), 34.75% (TBF), and 18.65% (BB3) ROS formation compared to the AAPH-stimulated solvent control in the highest tested concentration (Figure 6). No synergistic or antagonistic effects of the single components in the mixture could be observed.

### 3.6. Synergistic Induction of ARE-Dependent Gene Expression

**Figure 7:**
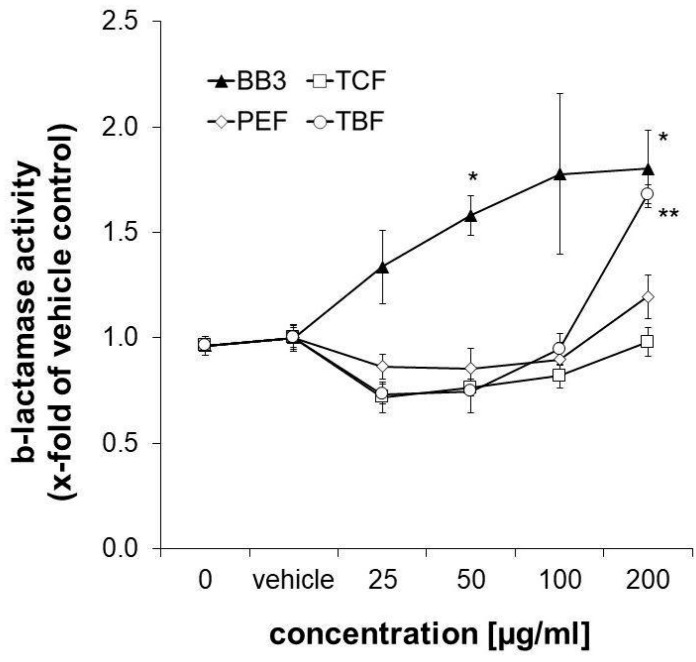
Influence of plant extracts on ß-lactamase activity, which was taken as a measure for ARE-dependent gene expression, in CellSensor^®^ ARE-bla HepG2 cells (expressed as n-fold to the corresponding control solvent). Shown are the mean values ± S.E.M. of three independent experiments with four parallels per concentration. (* *p* < 0.05 and ** *p* < 0.01; compared to vehicle-treated control cells).

Nrf-2/antioxidant response signalling was studied in the CellSensor^®^ ARE-bla HepG2 reporter liver cell line after 18 h of exposure to the extracts. β-Lactamase expression was taken as a measure for ARE-dependent gene expression. It was slightly decreased upon treatment with the three single components of BB3 in the lower concentration range (25–50 µg/mL), while the highest concentration of 200 µg/mL of TBF led to a significant increase by up to 1.68-fold compared to the control solvent. The same concentration of PEF induced an increase in ARE-dependent gene expression, however, this was non-significant and to a smaller extent (1.196-fold, as can be seen in Figure 7). A total of 200 µg/mL of the TCF extract, on the other hand, did not exhibit any significant effects (0.98-fold). Interestingly, the combination of the different herbal extracts with BB3 synergistically induced β-lactamase expression in a significant and dose-dependent manner by up to 1.80-fold at the highest tested concentration (200 µg/mL).

### 3.7. Gene Expression Changes

Gene expression changes in the LPS-stimulated THP-1 cells treated with BB3 extract (25 µg/mL) versus the vehicle control treatment were analysed using Affymetrix Human Genome U133 Plus 2.0 Arrays. The objective was to identify significant gene expression modifications post-treatment. Out of 20,857 unique genes annotated from the microarray data, 202 showed altered expression: 111 upregulated and 91 downregulated. Among these, 44 had a fold change greater than 2 (Supplementary Table S1).

#### 3.7.1. Pathway Enrichment Analysis with ClueGO

**Figure 8:**
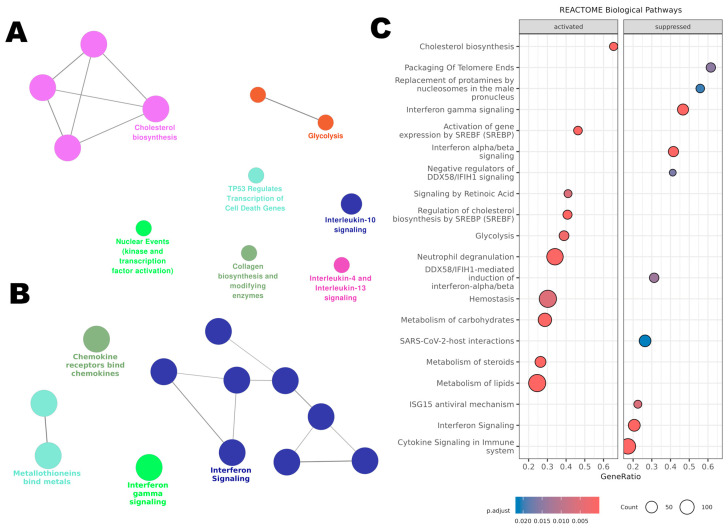
Affected pathways in THP-1 cells treated with BB3 were analysed using the Reactome database. (**A**) ClueGO enrichment analysis for genes that were upregulated post-treatment. (**B**) ClueGO analysis for the most significantly downregulated genes. (**C**) Gene Set Enrichment Analysis (GSEA) of Reactome pathways, including all genes organized by their expression changes as log2 fold change.

Using ClueGO for pathway enrichment analysis, the upregulated (Figure 8A) and downregulated (Figure 8B) gene lists were integrated with the Reactome database. For the upregulated genes, ClueGO revealed an activation of metabolic pathways. ‘Glycolysis’ was notably activated, suggesting a possible shift away from aerobic energy production. Pathways like ‘Cholesterol Biosynthesis’ were also upregulated, which could have implications for membrane structure and function. The upregulation of ‘Nuclear Events (Kinase and Transcription Factor Activation)’ suggests an activation in cellular proliferation and transcriptional activities.

Furthermore, there was an enrichment of pathways related to ‘Interleukin-10 Signaling’ and ‘Interleukin-4 and Interleukin-13 Signaling’, indicative of a possible anti-inflammatory or Th2-skewed immune response.

The downregulated genes clustered significantly within immune-related pathways. Notably, ‘Interferon Signaling’ and ‘Chemokine Receptors Bind Chemokines’ pathways were predominant, reflecting an inactivated state of the immune response. Additionally, ‘Metallothioneins Bind Metals’, indicating a potential decrease in cellular protective mechanisms against metal stress.

To identify the pathways influenced by BB3 extract, we conducted a Gene Set Enrichment Analysis (GSEA) using our microarray dataset and using the Reactome database. Through this analysis, 46 pathways emerged as statistically significant, each with an adjusted *p*-value below 0.05.

The top 20 pathways enriched among both upregulated and downregulated genes are shown in Figure 8C. For the pathways that were more active, the analysis highlighted those involved in ‘Cholesterol Biosynthesis’, ‘Regulation of Cholesterol Biosynthesis by SREBP’, ‘Lipid Metabolism’, ‘Glycolysis’, ‘Carbohydrate Metabolism’, ‘Blood Clotting’, and ‘Activation of Neutrophils’. On the other hand, the less active pathways included those related to ‘Telomere Maintenance’, ‘Interferon Gamma and Alpha/Beta Signaling’, ‘Regulation of DDX58/IFIH1 Signaling’, and the ‘ISG15 Antiviral Response’. All significant pathways identified are listed in Supplementary Table S1.

#### 3.7.2. Validation of Gene Expression

Gene expression results were validated for selected target genes in four independent experiments by using qPCR (n = 4). The LPS-stimulated cells were treated with 25 µg/mL (and 50 µg/mL) of BB3 for 24 h, and the expression was compared to the LPS treatment alone. Transcript selection for RT-qPCR analysis was performed by taking into consideration both microarray and previous functional assay results, as well as literature knowledge.

In Table 1, the gene regulation determined by RT-qPCR for the 25 µg/mL of BB3 treatment was compared to the results obtained in the microarray experiment (results expressed as fold changes) for 8 transcripts. C-X-C motif chemokine ligand 11 (*CXCL11*) and the signal transducer and activator of transcription 1 (*STAT1*) turned out to be significantly downregulated, and squalene epoxidase (*SQLE*) was found to be significantly upregulated (*p* < 0.05). These changes remained significant also with the BB3 treatment concentration of 50 µg/mL (fold changes (FCs) for *CXCL11* = 0.155, *SQLE* = 1.848 and *STAT1* = 0.445, all *p* < 0.05).

## 4. Discussion

Botanical remedies are highly valued in traditional systems of medicine for the treatment of a wide range of diseases, but little is known about the molecular mode of action. This study explored the effects of myrobalan fruit extracts on antioxidant and immunobiochemical pathways in order to get a better insight into their mode of action. The antioxidant capacities of PEF, TCF, TBF, and the mixture BB3 were confirmed both by a cell-free peroxyl radical scavenging assay and by measuring the intracellular ROS scavenging potential in the liver cell line HepG2 after stimulation with a peroxyl-radical generator. Remarkably, the values obtained in the ORAC assay of more than 2500 μmol TE/g indicate a strong antioxidant activity. ROS protection in cells accounts for some aspects of bioavailability of relevant constituents [40], and this effect was strongly dose dependent.

In addition to direct antioxidant properties, there are further mechanisms by which the intracellular antioxidant capacity can be strengthened [3]. This can occur, e.g., by activating signalling events that upregulate the expression of cytoprotective, detoxifying, and antioxidant genes, such as the Nrf-2/ARE system, an important signalling pathway that is activated to protect cells from oxidative and xenobiotic damage [41]. The activation of Nrf-2/ARE signalling was analysed by using the CellSensor^®^ ARE-bla HepG2 cells and showed a dose-dependent activation in the BB3 mixture. TBF treatment led to an effect only at the highest treatment concentration, and the other plant extracts were inactive. This may indicate a synergistic effect of the three myrobalan extracts on Nrf-2/ARE signalling. An increased expression of target genes such as *HMOX1* could be shown also in another cell model, THP-1 treated with LPS, as discussed below.

Interestingly, a time-dependent effect on NF-κB activity was observed, both in the LPS-stimulated and the unstimulated THP1-Blue^TM^ cells. After 6 h of treatment, a reduction in NF-κB activity was observed at higher concentrations in the stimulated cells—a slight decrease was also noted in the unstimulated cells. It was shown previously that Triphala herbal extract counteracted intracellular free radical formation and suppressed inflammatory responses in LPS-stimulated mouse RAW 264.7 macrophages by inhibition of the formation of pro-inflammatory mediators like TNF-α, IL-1β, IL-6, and nitric oxide [42]. However, in THP1-Blue^TM^, after 24 h, the treatment with all extracts led to a significant increase in NF-κB activity in the stimulated cells. The effect on the NF-κB signalling therefore appears to be strongly time and concentration dependent, which complicates the comprehensive assessment of this activity.

An additional indication of anti-inflammatory activity is the dose-dependent suppression of Trp breakdown and neopterin formation by the BB3 extracts in the stimulated THP-1 cells. The immunoregulatory enzyme IDO-1 is responsible for the conversion of Trp to Kyn, and neopterin is formed by the enzyme GTP-CH-I. Both signalling pathways are activated during the course of the cellular immune response and have been used as biomarkers in several in vitro and clinical settings. Importantly, the integration of IDO-1 and GTP-CH-I activity has previously been suggested as a screening tool for novel anti-inflammatory compounds [26]. As with the effect of BB3 on NF-κB activity, the effect varies in strength depending on the inflammatory status of the cell system. Furthermore, it remains to be investigated whether the observed effects are due to direct enzyme inhibition or whether the antioxidant effect of BB3 affects the signalling cascades that activate IDO-1 and GTP-CH-I activity.

The transcriptomic analysis further elucidated the mechanistic pathways influenced by myrobalan extracts. The LPS-stimulated THP-1 cells were treated with BB3 extract, and gene expression was analysed in comparison to the vehicle-treated LPS-stimulated cells. In total, 44 genes were found to have an FC higher than 2.

The observed downregulation of genes such as the C-X-C motif chemokine ligands *CXCL11*, *CXCL10*, *CXCL9*, *STAT1*, the genes of interferon inducible proteins *IFI27*, *IFI44*, and *IFIT2*, alongside the upregulation of certain other transcripts, underscores the potential of these extracts to mitigate inflammation and oxidative stress. Notably, chemokines like CXCL9, CXCL10, and CXCL11 are primarily produced by a variety of cell types, including monocytes, endothelial cells, fibroblasts, and cancer cells, in response to IFN-γ, and this response is further amplified by TNF-α, highlighting their role as pro-inflammatory cytokines [43]. These chemokines exert their effects through the activation of specific G protein-coupled receptors, facilitating the migration of both inflammatory and non-inflammatory cells to targeted tissues, thereby playing a pivotal role in immune regulation [44]. In terms of immune cell activation, CXCL9, CXCL10, and CXCL11 are known to promote Th1 cell polarization and activation [45]. Their downregulation in this study suggests an anti-inflammatory influence of BB3, potentially through the suppression of these IFN-inducible CXCR3 chemokines within the CXC-family. Furthermore, the notable downregulation of *STAT1*, a key transcription factor integral to mediating IFN-γ effects and broader cytokine responses, aligns with the immunomodulatory outcomes that were observed, reinforcing the hypothesis that myrobalan-based remedies may temper immune responses. This interpretation is supported by pathway analysis and ClueGo results, which revealed a downregulation in pathways related to ‘Chemokine Receptors Bind Chemokines’, consistent with previous findings that show oxidative stress can impair immune function by diminishing the expression and efficacy of the T-cell receptor complex [46,47].

The transcriptomic analysis revealed an upregulation of genes implicated in antioxidant responses, notably, early growth response (*EGR*)*1*, *EGR3*, N-myc downstream regulated (*NDRG*)*1*, and various regulator of G protein signaling (RGS) genes (RGS13, RGS1, and RGS16), with changes (FC) exceeding 2-fold. This upregulation underscores the pleiotropic nature of the myrobalan mixture BB3, which seems to orchestrate a multifaceted modulation of cellular pathways. The EGR family, particularly *EGR1*, is recognized for its expansive biological role in mediating physiological responses to stress, contributing to cellular growth and modulating tissue remodelling during pathological states, often induced by factors like transforming growth factor (TGF)-β and hypoxia [48]. *EGR3*, while less characterized, is known for its involvement in vascular endothelial growth factor (VEGF)-mediated angiogenesis, suggesting a role in cellular response mechanisms to oxidative stress and potentially contributing to the therapeutic angiogenic properties of Triphala [49].

As already mentioned above, the important role of Nrf-2 signalling in protecting cells against oxidative stress is highlighted by the increased activity of targets like heme oxygenase in HepG2 cells treated with the BB3 mixture. Although the fold change for *HMOX1* was modest at 1.3, it is a key enzyme in the oxidative stress defense. This may highlight the BB3 impact activating the cell’s endogenous antioxidative mechanisms. This moderate induction of *HMOX1*, coupled with the previously discussed upregulation of other antioxidant genes, supports the hypothesis that BB3 contributes to the reinforcement of the Nrf-2 pathway, subsequently promoting the expression of an array of antioxidative and phase II detoxifying enzymes.

Simultaneously, ClueGo found an upregulation of the ‘TP53 Regulates Transcription of Cell Death Genes’. In addition, the induction of NAD(P)H quinone dehydrogenase 1 (NQO1) via both the Nrf-2 and arylhydrocarbon receptor (AhR) pathway highlights a protective mechanism against oxidative hepatic damage [50], suggesting that BB3 might exert hepatoprotective effects. Moreover, it is recognized that NQO1 has the capability to stabilize the p53 tumour suppressor protein, a function that becomes particularly prominent under conditions of oxidative stress [51]. This is corroborated by the involvement of AhR in cellular metabolic regulation, particularly under stress conditions [52]. Importantly, Kyn as an endogenous ligand of AhR, is constitutively generated via the Trp catabolic pathway [53], as well as its catabolite kynurenic acid [54]. Such insights are further substantiated by the regulation of genes like 3-hydroxy-3-methylglutaryl-CoA synthase 1 (*HMGCS1*), which, as a pivotal enzyme in the mevalonate pathway, plays a dual role in both cholesterol synthesis and stress response, underscoring its potential as a pharmacological target in the context of acute myeloid leukemia (AML) chemoresistance [55].

There are several limitations of this in vitro study. The mode of action analysis of botanical remedies requires many considerations. On the one hand, due to the complex chemical composition of the mixture, there is only a limited potential to predict activities based on single active ingredients, and the perturbation of multiple molecular targets can be expected; on the other the hand, functional redundancy and regulatory loops on cell signalling levels add further complexity [1,4]. An important aspect is the choice of the cell system, and a disadvantage of cell lines is that they often differ strongly from the corresponding cell type in a healthy organism. On the other hand, specialized culture conditions may support gene expression at in vivo-like levels [56]. 

HepG2 is a popular hepatic cell line and is used for different purposes ranging from cancer research to cytotoxicity studies; however, the heterogeneity of the cell line and the weak or absent expression of certain cytochrome P450 (CYP) superfamily members compared to normal hepatocytes limits the predictive value of this model [57,58]. Nevertheless, it is believed that the HepG2 cell line has retained most of the metabolic functions of normal hepatocytes; therefore, it is a standard model for in vitro toxicology [59]. Also, the myelomonocytic cell line THP-1 which was derived from a leukaemia patient does not exhibit all characteristics of classically activated macrophages; however, it has more phenotypic similarities with monocytes than other myeloid cell lines [60]. Thus, there are limitations of the model systems that need to be considered; moreover, future studies need to include information on the interactions between different cell types and tissues. The limitations for extrapolating in vivo to in vitro findings are also based on the difficulties in simulating the consequences of long-term exposures in vitro [61]. Moreover, effects resulting from changing microbiomes or resulting from microbial-derived metabolic products are usually not considered. For example, Olenikov et al. reported that chebulic ellagitannins are transformed into bioaccessible colonic metabolites (urolithins) by gut microbiota [9] that readily enter systemic circulation and may contribute to systemic antioxidant effects [62].

All these aspects render the extrapolation from perturbed pathways or biomarkers in vitro to systemic adverse effects in vivo complicated. The limitations of in vitro models can be partially mitigated by integrating large-scale interaction databases, allowing researchers to construct more comprehensive models that capture the complexity of biological systems [63,64,65]. The application of strategies in which the combination of selected bioassays and computer models can support the investigation of mechanisms of action and increase the translational relevance of molecular analyses is of current interest.

To summarize, we could demonstrate that myrobalan extracts, as well as BB3, a mixture thereof, can modulate pathways that are involved in the regulation of inflammatory and cytoprotective processes in cell lines. Depending on the cell model and readout, both treatment time and dose were relevant for the type of interaction. The metabolic pathways investigated are also highly relevant for immunological and metabolic processes in vivo, and key molecules are used as in vivo biomarkers. However, more in-depth investigations are necessary for an in vitro to in vivo extrapolation of the results of this study. As suggested previously [1,5], the application of transcriptomics both supported, on the one hand, the results obtained by pathway-focused analysis and, on the other, revealed additional cellular processes and pathways that can be explored for a more detailed mode of action analysis. In addition, identified activity markers could also be used for the standardisation of extracts. Thus, although extrapolation of these findings to the in vivo situation is not possible, the results obtained provide a rationale for the design of further studies.

## 5. Conclusions

In conclusion, the findings of this study show an influence on signalling pathways that may provide an explanation for the effects of botanical extracts described in traditional systems of medicine. Furthermore, such studies can also be used to specify areas of application or to evaluate possible undesirable effects by providing a better understanding of molecular mode(s) of action. This supports a more comprehensive risk–benefit assessment.

## Figures and Tables

**Table 1:** Key genes modulated by the myrobalan mixture BB3 in lipopolysaccharide (LPS)-stimulated THP-1 cells. LPS-stimulated cells were treated with 25 µg/mL (and 50 µg/mL) of BB3, and the expression was compared to the LPS treatment alone. RT-qPCR relative expression ratios were normalized to *RPL37A* expression, and fold changes (FCs) are shown for the treatment with 25 µg/mL of extract.

		Microarray (n = 1)	RT-qPCR (n = 4)	
Gene	Gene Name	Log2FC	FC	*p*-Value
*IDO1*	Indoleamine 2,3-dioxygenase 1	−0.24	0.763	0.562
*CXCL11*	C-X-C motif chemokine ligand 11	−1.71	0.233	0.001
*SQLE*	Squalene epoxidase	0.61	1.581	0.011
*NFKB1*	Nuclear factor kappa B subunit 1	0.05	0.935	0.858
*HMGCR*	3-hydroxy-3-methylglutaryl-CoA reductase	0.63	1.377	0.478
*STAT1*	Signal transducer and activator of transcription 1	−0.56	0.507	0.002
*GCH*	GTP cyclohydrolase 1	−0.23	0.738	0.087
*TNF*	Tumor necrosis factor	−0.35	0.956	0.894

## Data Availability

Gene expression data are deposited at GEO (GSE273987). All other data are made available upon request.

## Supplementary Materials

The following supporting information can be downloaded at: https://www.mdpi.com/article/10.3390/antiox14030350/s1, Table S1: Complete list of probes, their annotated genes, and significant genes with a fold change (FC) higher than 1.

## References

[1] Gostner J.M., Wrulich O.A., Jenny M., Fuchs D., Ueberall F. (2012). An Update on the Strategies in Multicomponent Activity Monitoring within the Phytopharmaceutical Field. BMC Complement. Altern. Med..

[2] Wink M. (2008). Evolutionary Advantage and Molecular Modes of Action of Multi-Component Mixtures Used in Phytomedicine. Curr. Drug Metab..

[3] Dinkova-Kostova A.T., Talalay P. (2008). Direct and Indirect Antioxidant Properties of Inducers of Cytoprotective Proteins. Mol. Nutr. Food Res..

[4] Klein A., Wrulich O.A., Jenny M., Gruber P., Becker K., Fuchs D., Gostner J.M., Uberall F. (2013). Pathway-Focused Bioassays and Transcriptome Analysis Contribute to a Better Activity Monitoring of Complex Herbal Remedies. BMC Genom..

[5] Ulrich-Merzenich G., Panek D., Zeitler H., Wagner H., Vetter H. (2009). New Perspectives for Synergy Research with the “Omic”-Technologies. Phytomed. Int. J. Phytother. Phytopharm..

[6] Singh R., Singh B., Kumarb N., Arora S. (2010). Antioxidant Activity of Triphala a Combination of *Terminalia chebula*, *Terminalia bellerica* and *Emblica officinalis*. J. Food Biochem..

[7] Peterson C.T., Denniston K., Chopra D. (2017). Therapeutic Uses of Triphala in Ayurvedic Medicine. J. Altern. Complement. Med..

[8] Long X.-M., Li R., Liu H.-P., Xia Z.-X., Guo S., Gu J.-X., Zhang L.-J., Fan Y., Chen Z.-K. (2023). Chemical Fingerprint Analysis and Quality Assessment of Tibetan Medicine Triphala from Different Origins by High-Performance Liquid Chromatography. Phytochem. Anal..

[9] Olennikov D.N., Kashchenko N.I., Chirikova N.K. (2015). In Vitro Bioaccessibility, Human Gut Microbiota Metabolites and Hepatoprotective Potential of Chebulic Ellagitannins: A Case of Padma Hepaten^®^ Formulation. Nutrients.

[10] Tarasiuk A., Mosińska P., Fichna J. (2018). Triphala: Current Applications and New Perspectives on the Treatment of Functional Gastrointestinal Disorders. Chin. Med..

[11] Naik G.H., Priyadarsini K.I., Bhagirathi R.G., Mishra B., Mishra K.P., Banavalikar M.M., Mohan H. (2005). In Vitro Antioxidant Studies and Free Radical Reactions of Triphala, an Ayurvedic Formulation and Its Constituents. Phytother. Res..

[12] Kaur S., Michael H., Arora S., Härkönen P.L., Kumar S. (2005). The in Vitro Cytotoxic and Apoptotic Activity of Triphala—An Indian Herbal Drug. J. Ethnopharmacol..

[13] Gostner J.M., Becker K., Fuchs D., Sucher R. (2013). Redox Regulation of the Immune Response. Redox Rep. Commun. Free Radic. Res..

[14] Werner E.R., Werner-Felmayer G., Fuchs D., Hausen A., Reibnegger G., Wachter H. (1989). Parallel Induction of Tetrahydrobiopterin Biosynthesis and Indoleamine 2,3-Dioxygenase Activity in Human Cells and Cell Lines by Interferon-Gamma. Biochem. J..

[15] Saha B., Jyothi Prasanna S., Chandrasekar B., Nandi D. (2010). Gene Modulation and Immunoregulatory Roles of Interferon Gamma. Cytokine.

[16] Hatano E., Bennett B.L., Manning A.M., Qian T., Lemasters J.J., Brenner D.A. (2001). NF-kappaB Stimulates Inducible Nitric Oxide Synthase to Protect Mouse Hepatocytes from TNF-Alpha- and Fas-Mediated Apoptosis. Gastroenterology.

[17] Jaiswal A.K. (2004). Nrf2 Signaling in Coordinated Activation of Antioxidant Gene Expression. Free Radic. Biol. Med..

[18] Funes S.C., Rios M., Fernández-Fierro A., Covián C., Bueno S.M., Riedel C.A., Mackern-Oberti J.P., Kalergis A.M. (2020). Naturally Derived Heme-Oxygenase 1 Inducers and Their Therapeutic Application to Immune-Mediated Diseases. Front. Immunol..

[19] Kapturczak M.H., Wasserfall C., Brusko T., Campbell-Thompson M., Ellis T.M., Atkinson M.A., Agarwal A. (2004). Heme Oxygenase-1 Modulates Early Inflammatory Responses: Evidence from the Heme Oxygenase-1-Deficient Mouse. Am. J. Pathol..

[20] Fallarino F., Grohmann U., Vacca C., Bianchi R., Orabona C., Spreca A., Fioretti M.C., Puccetti P. (2002). T Cell Apoptosis by Tryptophan Catabolism. Cell Death Differ..

[21] Fuchs D., Malkovsky M., Reibnegger G., Werner E.R., Forni G., Wachter H. (1989). Endogenous Release of Interferon-Gamma and Diminished Response of Peripheral Blood Mononuclear Cells to Antigenic Stimulation. Immunol. Lett..

[22] Taylor M.W., Feng G.S. (1991). Relationship between Interferon-Gamma, Indoleamine 2,3-Dioxygenase, and Tryptophan Catabolism. FASEB J..

[23] Schröcksnadel K., Wirleitner B., Winkler C., Fuchs D. (2006). Monitoring Tryptophan Metabolism in Chronic Immune Activation. Clin. Chim. Acta Int. J. Clin. Chem..

[24] Murr C., Widner B., Wirleitner B., Fuchs D. (2002). Neopterin as a Marker for Immune System Activation. Curr. Drug Metab..

[25] Jenny M., Klieber M., Zaknun D., Schroecksnadel S., Kurz K., Ledochowski M., Schennach H., Fuchs D. (2011). In Vitro Testing for Anti-Inflammatory Properties of Compounds Employing Peripheral Blood Mononuclear Cells Freshly Isolated from Healthy Donors. Inflamm. Res..

[26] Becker K., Schwaiger S., Waltenberger B., Fuchs D., Pezzei C.K., Schennach H., Stuppner H., Gostner J.M. (2018). Immunomodulatory Effects of Diterpene Quinone Derivatives from the Roots of Horminum Pyrenaicum in Human PBMC. Oxidative Med. Cell. Longev..

[27] Plaskova A., Mlcek J. (2023). New Insights of the Application of Water or Ethanol-Water Plant Extract Rich in Active Compounds in Food. Front. Nutr..

[28] Ou B., Hampsch-Woodill M., Prior R.L. (2001). Development and Validation of an Improved Oxygen Radical Absorbance Capacity Assay Using Fluorescein as the Fluorescent Probe. J. Agric. Food Chem..

[29] Jenny M., Santer E., Klein A., Ledochowski M., Schennach H., Ueberall F., Fuchs D. (2009). Cacao Extracts Suppress Tryptophan Degradation of Mitogen-Stimulated Peripheral Blood Mononuclear Cells. J. Ethnopharmacol..

[30] Widner B., Werner E.R., Schennach H., Wachter H., Fuchs D. (1997). Simultaneous Measurement of Serum Tryptophan and Kynurenine by HPLC. Clin. Chem..

[31] Geisler S., Mayersbach P., Becker K., Schennach H., Fuchs D., Gostner J.M. (2015). Serum Tryptophan, Kynurenine, Phenylalanine, Tyrosine and Neopterin Concentrations in 100 Healthy Blood Donors. Pteridines.

[32] Zlokarnik G., Negulescu P.A., Knapp T.E., Mere L., Burres N., Feng L., Whitney M., Roemer K., Tsien R.Y. (1998). Quantitation of Transcription and Clonal Selection of Single Living Cells with β-Lactamase as Reporter. Science.

[33] Bass D.A., Parce J.W., Dechatelet L.R., Szejda P., Seeds M.C., Thomas M. (1983). Flow Cytometric Studies of Oxidative Product Formation by Neutrophils: A Graded Response to Membrane Stimulation. J. Immunol..

[34] Wang H., Joseph J.A. (1999). Quantifying Cellular Oxidative Stress by Dichlorofluorescein Assay Using Microplate Reader. Free Radic. Biol. Med..

[35] Pfaffl M.W. (2001). A New Mathematical Model for Relative Quantification in Real-Time RT-PCR. Nucleic Acids Res..

[36] Yu G., He Q.-Y. (2016). ReactomePA: An R/Bioconductor Package for Reactome Pathway Analysis and Visualization. Mol. Biosyst..

[37] Bindea G., Mlecnik B., Hackl H., Charoentong P., Tosolini M., Kirilovsky A., Fridman W.-H., Pagès F., Trajanoski Z., Galon J. (2009). ClueGO: A Cytoscape Plug-in to Decipher Functionally Grouped Gene Ontology and Pathway Annotation Networks. Bioinformatics.

[38] Schroecksnadel S., Jenny M., Fuchs D. (2011). Myelomonocytic THP-1 Cells for In Vitro Testing of Immunomodulatory Properties of Nanoparticles. J. Biomed. Nanotechnol..

[39] Schroecksnadel S., Jenny M., Kurz K., Klein A., Ledochowski M., Überall F., Fuchs D. (2010). LPS-Induced NF-κB Expression in THP-1Blue Cells Correlates with Neopterin Production and Activity of Indoleamine 2,3-Dioxygenase. Biochem. Biophys. Res. Commun..

[40] Wolfe K.L., Liu R.H. (2007). Cellular antioxidant activity (CAA) assay for assessing antioxidants, foods, and dietary supplements. J. Agric. Food Chem..

[41] Niture S.K., Khatri R., Jaiswal A.K. (2014). Regulation of Nrf2-an update. Free Radic. Biol. Med..

[42] Kalaiselvan S., Rasool M.K. (2016). Triphala Herbal Extract Suppresses Inflammatory Responses in LPS-Stimulated RAW 264.7 Macrophages and Adjuvant-Induced Arthritic Rats via Inhibition of NF-κB Pathway. J. Immunotoxicol..

[43] Ohmori Y., Schreiber R.D., Hamilton T.A. (1997). Synergy between Interferon-Gamma and Tumor Necrosis Factor-Alpha in Transcriptional Activation Is Mediated by Cooperation between Signal Transducer and Activator of Transcription 1 and Nuclear Factor kappaB. J. Biol. Chem..

[44] Fernandez E.J., Lolis E. (2002). Structure, Function, and Inhibition of Chemokines. Annu. Rev. Pharmacol. Toxicol..

[45] Tokunaga R., Zhang W., Naseem M., Puccini A., Berger M.D., Soni S., McSkane M., Baba H., Lenz H.-J. (2018). CXCL9, CXCL10, CXCL11/CXCR3 Axis for Immune Activation—A Target for Novel Cancer Therapy. Cancer Treat. Rev..

[46] Saccani A., Saccani S., Orlando S., Sironi M., Bernasconi S., Ghezzi P., Mantovani A., Sica A. (2000). Redox Regulation of Chemokine Receptor Expression. Proc. Natl. Acad. Sci. USA.

[47] Otsuji M., Kimura Y., Aoe T., Okamoto Y., Saito T. (1996). Oxidative Stress by Tumor-Derived Macrophages Suppresses the Expression of CD3 Zeta Chain of T-Cell Receptor Complex and Antigen-Specific T-Cell Responses. Proc. Natl. Acad. Sci. USA.

[48] Thiel G., Cibelli G. (2002). Regulation of Life and Death by the Zinc Finger Transcription Factor Egr-1. J. Cell. Physiol..

[49] Suehiro J., Hamakubo T., Kodama T., Aird W.C., Minami T. (2010). Vascular Endothelial Growth Factor Activation of Endothelial Cells Is Mediated by Early Growth Response-3. Blood.

[50] Dong H., Hao L., Zhang W., Zhong W., Guo W., Yue R., Sun X., Zhou Z. (2021). Activation of AhR-NQO1 Signaling Pathway Protects Against Alcohol-Induced Liver Injury by Improving Redox Balance. Cell. Mol. Gastroenterol. Hepatol..

[51] Asher G., Lotem J., Kama R., Sachs L., Shaul Y. (2002). NQO1 Stabilizes P53 through a Distinct Pathway. Proc. Natl. Acad. Sci. USA.

[52] Terashima J., Habano W., Gamou T., Ozawa S. (2011). Induction of CYP1 Family Members under Low-Glucose Conditions Requires AhR Expression and Occurs through the Nuclear Translocation of AhR. Drug Metab. Pharmacokinet..

[53] Opitz C.A., Litzenburger U.M., Sahm F., Ott M., Tritschler I., Trump S., Schumacher T., Jestaedt L., Schrenk D., Weller M. (2011). An Endogenous Tumour-Promoting Ligand of the Human Aryl Hydrocarbon Receptor. Nature.

[54] DiNatale B.C., Murray I.A., Schroeder J.C., Flaveny C.A., Lahoti T.S., Laurenzana E.M., Omiecinski C.J., Perdew G.H. (2010). Kynurenic Acid Is a Potent Endogenous Aryl Hydrocarbon Receptor Ligand That Synergistically Induces Interleukin-6 in the Presence of Inflammatory Signaling. Toxicol. Sci..

[55] Zhou C., Li J., Du J., Jiang X., Xu X., Liu Y., He Q., Liang H., Fang P., Zhan H. (2021). HMGCS1 Drives Drug-Resistance in Acute Myeloid Leukemia through Endoplasmic Reticulum-UPR-Mitochondria Axis. Biomed. Pharmacother..

[56] Ghallab A. (2013). In Vitro Test Systems and Their Limitations. EXCLI J..

[57] Arzumanian V.A., Kiseleva O.I., Poverennaya E.V. (2021). The Curious Case of the HepG2 Cell Line: 40 Years of Expertise. Int. J. Mol. Sci..

[58] Guengerich F.P. (2019). Cytochrome P450 Research and The Journal of Biological Chemistry. J. Biol. Chem..

[59] Pareek A., Godavarthi A., Issarani R., Nagori B.P. (2013). Antioxidant and Hepatoprotective Activity of *Fagonia schweinfurthii* (Hadidi) Hadidi Extract in Carbon Tetrachloride Induced Hepatotoxicity in HepG2 Cell Line and Rats. J. Ethnopharmacol..

[60] Baxter E.W., Graham A.E., Re N.A., Carr I.M., Robinson J.I., Mackie S.L., Morgan A.W. (2020). Standardized Protocols for Differentiation of THP-1 Cells to Macrophages with Distinct M(IFNγ+LPS), M(IL-4) and M(IL-10) Phenotypes. J. Immunol. Methods.

[61] Tice R.R., Austin C.P., Kavlock R.J., Bucher J.R. (2013). Improving the Human Hazard Characterization of Chemicals: A Tox21 Update. Environ. Health Perspect..

[62] Bialonska D., Kasimsetty S.G., Khan S.I., Ferreira D. (2009). Urolithins, Intestinal Microbial Metabolites of Pomegranate Ellagitannins, Exhibit Potent Antioxidant Activity in a Cell-Based Assay. J. Agric. Food Chem..

[63] Chen X., Yan C.C., Zhang X., Zhang X., Dai F., Yin J., Zhang Y. (2016). Drug-Target Interaction Prediction: Databases, Web Servers and Computational Models. Brief. Bioinform..

[64] Cidem A., Bradbury P., Traini D., Ong H.X. (2020). Modifying and Integrating in Vitro and Ex Vivo Respiratory Models for Inhalation Drug Screening. Front. Bioeng. Biotechnol..

[65] Kuenzi B.M., Park J., Fong S.H., Sanchez K.S., Lee J., Kreisberg J.F., Ma J., Ideker T. (2020). Predicting Drug Response and Synergy Using a Deep Learning Model of Human Cancer Cells. Cancer Cell.

